# Characterization of meiotic recombination intermediates through gene knockouts in founder hybrid mice

**DOI:** 10.1101/gr.278024.123

**Published:** 2023-11

**Authors:** Benjamin Davies, Gang Zhang, Daniela Moralli, Samy Alghadban, Daniel Biggs, Chris Preece, Peter Donnelly, Anjali Gupta Hinch

**Affiliations:** 1 Wellcome Centre for Human Genetics, University of Oxford, Oxford OX3 7BN, United Kingdom;; 2 The Francis Crick Institute, London NW1 1AT, United Kingdom;; 3 Genomics PLC, Oxford OX1 1JD, United Kingdom

## Abstract

Mammalian meiotic recombination proceeds via repair of hundreds of programmed DNA double-strand breaks, which requires choreographed binding of RPA, DMC1, and RAD51 to single-stranded DNA substrates. High-resolution in vivo binding maps of these proteins provide insights into the underlying molecular mechanisms. When assayed in F_1_-hybrid mice, these maps can distinguish the broken chromosome from the chromosome used as template for repair, revealing more mechanistic detail and enabling the structure of the recombination intermediates to be inferred. By applying CRISPR-Cas9 mutagenesis directly on F_1_-hybrid embryos, we have extended this approach to explore the molecular detail of recombination when a key component is knocked out. As a proof of concept, we have generated hybrid biallelic knockouts of *Dmc1* and built maps of meiotic binding of RAD51 and RPA in them. DMC1 is essential for meiotic recombination, and comparison of these maps with those from wild-type mice is informative about the structure and timing of critical recombination intermediates. We observe redistribution of RAD51 binding and complete abrogation of D-loop recombination intermediates at a molecular level in *Dmc1* mutants. These data provide insight on the configuration of RPA in D-loop intermediates and suggest that stable strand exchange proceeds via multiple rounds of strand invasion with template switching in mouse. Our methodology provides a high-throughput approach for characterization of gene function in meiotic recombination at low animal cost.

Homologous recombination (HR) in meiosis is an essential process underlying the production of gametes in sexually reproducing species and ensures the correct segregation of chromosomes into daughter cells (Hunter 2015). Recombination begins with the induction of programmed DNA double-strand breaks (DSBs), which may be repaired with reciprocal exchange of genetic material between homologous chromosomes, known as crossover, or partial segmental exchange, known as noncrossover (Baudat et al. 2013). This process is mediated via a complex choreography of proteins involved in the creation and repair of DSBs (Li et al. 2021). Despite significant recent progress, fundamental aspects of repair of these breaks remain unknown, especially in mammals.

Programmed DSBs in many organisms occur in discrete positions in the genome, known as “hotspots” (Baudat et al. 2013), which are positioned by the binding properties of the zinc-finger protein PRDM9 in the mouse, human, and many other vertebrates (Baudat et al. 2010; Myers et al. 2010; Parvanov et al. 2010). After the induction of a DSB, the broken ends are resected, and the resulting single-stranded DNA (ssDNA) is thought to be stabilized by the main eukaryotic ssDNA-binding protein RPA (Brown and Bishop 2014; Crickard and Greene 2018). RPA is replaced by the mammalian RecA orthologs, DMC1 and RAD51, which bind ssDNA to form nucleoprotein filaments (Fig. 1A; Crickard and Greene 2018). *Dmc1* is expressed only in meiotic cells and is essential for DSB repair and chromosomal synapsis during meiosis in mammals (Pittman et al. 1998; Yoshida et al. 1998). In contrast, RAD51 is expressed widely, including in non-meiotic cells, and is necessary for DSB repair by HR (Brown and Bishop 2014). Accordingly, loss of RAD51 in mouse results in an early embryonic lethality phenotype (Lim and Hasty 1996; Tsuzuki et al. 1996). RAD51 performs meiotic strand exchange in *Caenorhabditis elegans* (Woglar and Villeneuve 2018) and *Schizosaccharomyes pombe* (Murayama et al. 2008) and is necessary for meiosis in *Saccharomyces cerevisiae* (Cloud et al. 2012*)* and *Arabidopsis thaliana* (Da Ines et al. 2013a). However, its ability to perform strand exchange is dispensable in *S. cerevisiae* (Bishop 2012; Cloud et al. 2012; Da Ines et al. 2013b; Singh et al. 2017). RAD51 promotes the formation of nucleoprotein filaments (Bishop 2012; Chan et al. 2019) while also apparently competing with DMC1 for binding to the filament (Crickard et al. 2018). These filaments search for and invade the homologous chromosome to serve as templates for break repair (Crickard and Greene 2018). Numerous such sites of stable strand exchange between the homologous DNA molecules result in full pairing of the homologous chromosomes (Hunter 2015). In the absence of DMC1, RAD51 can perform strand exchange in particular *S. cerevisiae* mutants and in *A. thaliana*, although synapsis and segregation of homologous chromosomes remain defective (Da Ines et al. 2012; Lao et al. 2013).

**Figure 1.**
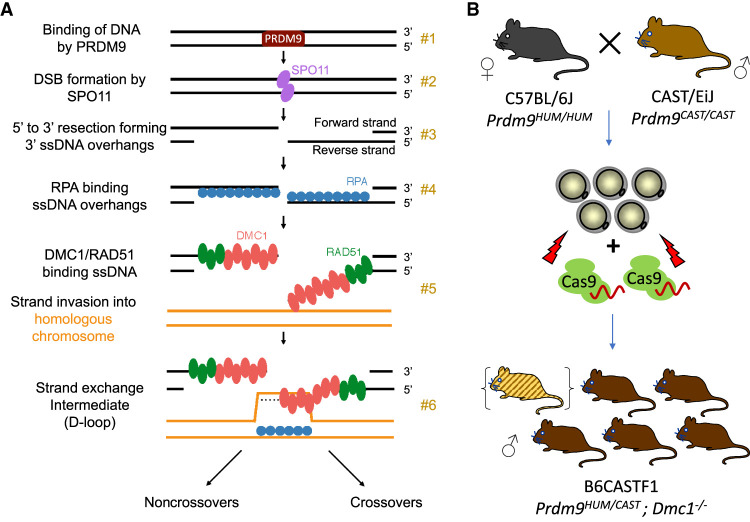
Methodology of mapping recombination intermediates using *Dmc1* knockout hybrid founders. (*A*) Key steps in mammalian meiotic recombination. PRDM9 binds DNA at particular sequence motifs (#1), a subset of which become sites of programmed DSBs by SPO11 (#2). Strand resection creates ssDNA overhangs, which are bound by RPA first (#3) and then by DMC1 and RAD51 (#4). Binding occurs to the left of the break on the forward strand and to the right of the break on the reverse strand. A segment of DMC1-bound DNA pairs with a strand on the homologous chromosome, forming a D-loop intermediate (#5, #6). (*B*) Production scheme of hybrid B6CASTF1 *Dmc1*^−/−^ mice. The *top* panel shows the parental cross, as well as the harvesting and electroporation of the F_1_ zygotes with CRISPR-Cas9 ribonucleoprotein targeting the *Dmc1* gene. Offspring are putative biallelic knockout founders. DMC1 immunohistochemical analysis allows founders with mosaic or remnant DMC1 to be excluded from the analysis (represented as a hybrid founder mouse in parenthesis).

We showed recently that single-stranded DNA sequencing (SSDS) assays for RPA, DMC1, and RAD51 together provide detailed quantitative and kinetic information on aspects of DSB processing, time taken to engage with the homolog used for repair, and the structure and lifespan of recombination intermediates (Hinch et al. 2020). These assays are most informative when performed in hybrid mice with significant heterozygosity in their chromosomes. This is because key recombination proteins bind the chromosome on which the DSB occurs (henceforth, DSB-initiating chromosome) and the homolog used as repair template (henceforth, repair-template chromosome) at different times. Mapping of protein binding in hybrid F_1_ mice, generated by crossing two strains with high sequence divergence, enables localization of events specifically to one or the other of the two homologs (Baker et al. 2015; Davies et al. 2016; Smagulova et al. 2016; Hinch et al. 2019, 2020; Li et al. 2019; Gergelits et al. 2021). The ability to extend this approach to genetically modified mice is a tool of obvious power, as it could illuminate the consequences of loss of function of key meiotic genes genome-wide, specifically and separately for sites in which DSBs take place and sites used as templates for repair.

Generating a homozygous gene loss-of-function mutant while preserving a clean F_1_ hybrid genetic background can be achieved using conventional technology for “knocking out” a gene in one of two ways, both with significant drawbacks. The standard approach is to generate a mutation on one parental background, and then the mutant allele is moved to the other background by sequential backcrossing. The original and the backcrossed strain are then bred together to generate an F_1_ knockout. Generating a sufficiently clean F_1_ hybrid is essential, as previous research has shown that a homozygous segment as small as 20–30 Mb can alter the recombination dynamics of the entire chromosome (Gregorova et al. 2018). Numerous backcross generations would be required, necessitating a large multiyear breeding program for each gene of interest.

An alternative possibility is to generate mutations separately on the two parental backgrounds, to interbreed heterozygous mice of each background, and then select the resulting F_1_ mice that have inherited two loss-of-function alleles. This approach requires the generation of genetic mutants on sufficiently diverged *Mus musculus* subspecies (e.g., *Mus musculus domesticus* and *Mus musculus castaneus* or *Mus musculus musculus*). Routine culture and genetic manipulation conditions for *M. musculus castaneus* and *M. musculus musculus* zygotes are not fully established, making this approach challenging. Both methods also suffer from the unexpected drawback that the high efficiency of CRISPR-Cas9 genome-editing mutagenesis results in a high rate of biallelic mutation (Wang et al. 2013). Because knocking out meiotic genes frequently leads to infertility, the founder mice cannot be bred to establish lines.

To overcome these challenges, we propose a methodology based on founder phenotype analysis: genetic modification of hybrid mice by CRISPR-Cas9 mutagenesis and selection of founders harboring biallelic loss-of-function mutations, followed by direct phenotyping using genome-wide assays measuring the occupancy of key meiotic repair proteins. As a proof of principle, we investigate the role of DMC1 using hybrid knockout mice. We chose this gene because the function of DMC1 is known and because *Dmc1* mutants on a homozygous background have been studied extensively (Pittman et al. 1998; Yoshida et al. 1998; Paiano et al. 2020; Yamada et al. 2020), providing us with a benchmark for validating our method. Although *Dmc1*^−/−^ male mice undergo DSBs and load RAD51 with normal kinetics, the breaks remain unrepaired, and the chromosomes fail to synapse, which leads to meiotic arrest.

The aims of this study were, first, to establish and validate an efficient knockout strategy of meiotic genes in hybrid F_1_ mice generated from crosses between a *M. musculus domesticus* mother and a *M. musculus castaneus* father and, second, to use SSDS to assess the binding positions and lifespans of RAD51 and RPA in the testes of these *Dmc1* knockout hybrids to further our understanding of the molecular structures involved in meiotic DNA break repair.

## Results

### Optimization of mutagenesis and validation of *Dmc1* knockouts

We performed mutagenesis on B6CASTF1 hybrid embryos, generated from parental mice of two subspecies with different *Prdm9* alleles: a previously described genetically altered C57BL/6J strain harboring humanized *Prdm9* (*Prdm9*^*HUM*^) (Davies et al. 2016) and wild-type *Mus castaneus* harboring a naturally occurring *Prdm9* allele (*Prdm9*^*CAST*^) (Fig. 1B). To explore the efficiency of achieving a functional knockout of *Dmc1*, we designed CRISPR-Cas9 site-specific nucleases initially against exons 6 and 7, encoding part of the AAA + ATPase domain, essential for catalytic function of the recombinase. The selected CRISPR-Cas9 target sites avoided known polymorphisms between C57BL/6J and CAST/EiJ. Mutagenesis using two CRISPR-Cas9 nucleases in *cis* would maximize the chances of a knockout, by either the introduction of frame-shifting indels at one or both of the exonic target sites or by deletion of critical coding sequence between the target sites.

We tested the single-guide RNAs (sgRNAs) for activity in vitro by assessing indel frequency in a mouse melanocyte cell line (B16F12 cells) and selected the highest performing sgRNAs (sgRNAs-A and sgRNA-D) for mutagenesis (Supplemental Fig. S1). These two sgRNAs were complexed with Cas9 protein, electroporated into fertilized B6CASTF1 hybrid zygotes, transferred to recipient foster females, and carried to term.

Of 11 pups born (five males and six females), all but one showed indel mutations at the 5′ CRISPR-Cas9 cut site (sgRNA-A), as determined by Sanger sequencing of an amplicon spanning the target sequence (Supplemental Table S1). Two of the pups born showed an in *cis* deletion between the cut sites (sgRNA-A and sgRNA-D), but amplicon sequencing at the 3′ CRISPR-Cas9 cut site (sgRNA-D) failed to detect any indel mutations. To check whether functional knockouts were generated, we validated the knockout status of the five male founders by immunohistochemistry with a DMC1-specific antibody on meiotic chromosome spreads.

**Figure 2.**
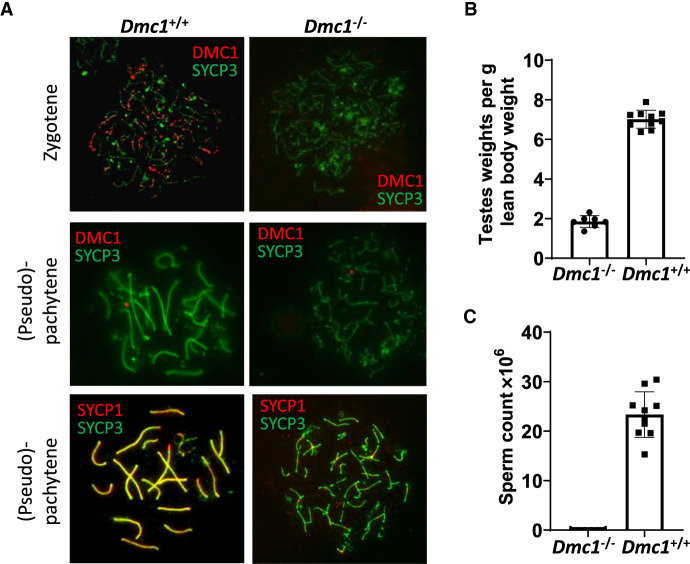
Investigation of fertility phenotypes of *Dmc1* hybrid founder mice. (*A*) Representative immunofluorescence staining of DMC1 (red) in testis nuclear spreads from two founder mice, one with DMC1 present (*Dmc1*^*+/+*^) and with DMC1 ablated (*Dmc1*^−/−^) with costaining of the synaptonemal complex protein SYCP3, which labels the chromosome axis (green) in zygotene (*top* panels) and pachytene/pseudo-pachytene (*middle* panels). *Lower* panels show pachytene/pseudo-pachytene stage staining of SYCP1 (red), labeling the synaptonemal-complex transverse filament, and SYCP3 (green) staining of the chromosome axes. Pronounced asynapsis is visible in a founder with ablated DMC1 (*Dmc1*^−/−^). (*B*,*C*) Fertility parameters for the founder mice showing complete ablation of DMC1 (*Dmc1*^−/−^; n = 7) with age-matched wild-type B6CASTF1 mice (*Dmc1*^+/+^; n = 10), with normalized testis weight (*B*) and total caudal sperm count (*C*).

One male founder (4.1a) revealed no evidence of *Dmc1* mutagenesis by sequencing (Supplemental Table S1) and showed a normal pattern of DMC1 staining, with the majority of staining at zygotene (Fig. 2A; Table 1). Three of the mutated males revealed a complete absence of DMC1 foci and no normal pachytene-stage cells (Fig. 2A), consistent with a functional knockout of *Dmc1*. One mutant male (3.1b) showed mosaic *Dmc1* expression (Table 1). Although clearly meiosis was disrupted in this animal, a low level of normal pachytene cells was observed, indicating that this mouse was a mosaic of mutant and wild-type cells.

**Table 1.**
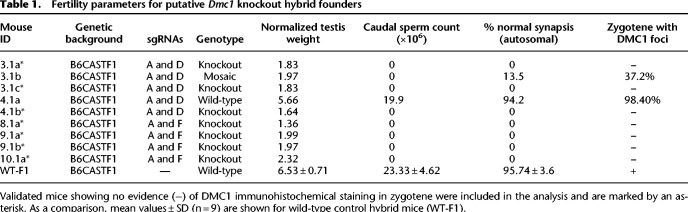
Fertility parameters for putative *Dmc1* knockout hybrid founders

Given the low level of mutagenesis at the 3′ CRISPR site using sgRNA-D and the mosaic expression of *Dmc1* observed in one of the founders, we wanted to improve the reliability of generating a knockout allele. We redesigned the mutagenesis strategy and tested an alternative 3′ CRISPR-Cas9 sgRNA (sgRNA-F), which binds within exon 8 with an aim of increasing the likelihood of mutagenesis (Supplemental Fig. S1). This sgRNA showed a high rate of mutagenesis in vitro, as assessed by a high frequency of indel mutations when introduced into a mouse melanocyte cell line (B16F12 cells) (Supplemental Fig. S1) and when tested in C57BL/6J embryos (Supplemental Table S2), and was used in combination with sgRNA-A for further mutagenesis experiments in mouse embryos. Electroporation of hybrid embryos using these optimized sgRNA combinations showed improved levels of mutagenesis with all of the seven resulting founder mice (four males and three females) showing mutagenesis at either the 5′, 3′, or both sites (Supplemental Table S1). Furthermore, two out of the seven founder mice generated using these optimized sgRNAs revealed the presence of an in *cis* deletion event between the two CRISPR-Cas9 sites (sgRNA-A and sgRNA-F). An investigation of DMC1 protein expression in the four putative hybrid knockout male mice showed a complete absence of DMC1 foci and no normal pachytene cells, suggesting that no mosaic expression of *Dmc1* occurred in testes of mutants generated with this optimized mutagenesis strategy (Table 1). Analysis of RPA foci by immunohistochemistry revealed an elevated number of foci in zygotene and pachytene, consistent with the persistence of unrepaired recombination intermediates (Supplemental Fig. S2; Pittman et al. 1998; Yoshida et al. 1998).

All founders were analyzed for sperm count and testes weight (Table 1). Founders that were validated as having a complete ablation of DMC1 by immunocytochemistry also showed a pronounced reduction in testes weight, consistent with the meiotic phenotype (Fig. 2B). Furthermore, no sperm could be isolated from their caudal epididymides (Fig. 2C).

### Genome-wide maps of RAD51 and RPA binding in *Dmc1*^−/−^ mouse testes

Using this pipeline of optimized mutagenesis via CRISPR-Cas9 electroporation, followed by verification of absence of DMC1 at the protein level, we generated maps of in vivo binding of RPA and RAD51 in candidate knockout founder mice. Specifically, we performed chromatin immunoprecipitation (ChIP) followed by ssDNA sequencing (ChIP-seq) (Khil et al. 2012) for RAD51 and RPA (Hinch et al. 2020) in testes of the adult male *Dmc1*^−/−^ hybrid mice generated above. *Dmc1*^−/−^ mice have small testes (Fig. 2B), and some of the testis tissue of each mouse was used for cytology, as described above. To ensure sufficient material for ChIP-seq experiments, we pooled tissue from three of the validated founder mice for each assay (3.1a, 3.1b, and 4.1b for RAD51 and 8.1a, 9.1a, and 9.1b for RPA). We observed that RAD51 and RPA ChIP-seq reads cluster into small regions that match known recombination hotspots identified by mapping of these proteins in *Dmc1*^*+/+*^ mice with the same genetic background (Hinch et al. 2019), which is consistent with the role of DMC1 being downstream from break formation. The signals of RAD51 and RPA in these hotspots have strong correlation with those in *Dmc1*^*+/+*^ mice (Pearson correlation coefficient *r* = 0.93 and *r* = 0.96, respectively, for autosomal hotspots). We identified 9023 and 13,348 peaks of RAD51 and RPA in *Dmc1*^−/−^ de novo, the vast majority of which overlapped previously identified hotspots (97% and 99%, respectively). Recombination hotspots in the hybrid mouse comprise hotspots that are activated by PRDM9^CAST^ or PRDM9^HUM^ and those that are PRDM9-independent (Hinch et al. 2019). PRDM9^CAST^ is dominant over PRDM9^HUM^ and PRDM9-independent autosomal hotspots for both the RAD51 (65% PRDM9^CAST^, 29% PRDM9^HUM^, 0% PRDM9-independent, 7% uncertain) and RPA assays (64% PRDM9^CAST^, 29% PRDM9^HUM^, 0% PRDM9-independent, 7% uncertain) to an extent consistent with *Dmc1*^*+/+*^ mice. Finally, we mapped ChIP-seq reads separately to the C57BL6/J (hereafter B6) or CAST/EiJ (hereafter CAST) haplotypes to generate homolog-specific maps for each protein. Examples of the RPA and DMC1 binding at individual hotspots are shown in Supplemental Figures S3 and S4.

### Characterization of changes in recombination intermediates in the absence of DMC1

Recombination hotspots in hybrid mice often vary in their activity on the two homologous chromosomes, usually as a result of differences in PRDM9 binding owing to sequence polymorphisms between them (Davies et al. 2016). Hotspots with similar levels of PRDM9 binding on both homologs are referred to as symmetric (Davies et al. 2016). In contrast, hotspots in which DSBs occur on only one of the B6 or CAST homologs are known as asymmetric hotspots. We use them to distinguish binding of RPA and RAD51 to the DSB-initiating chromosome from the repair-template chromosome by using the homolog-specific ChIP-seq maps generated above. We compared and contrasted binding patterns in *Dmc1*^−/−^ and *Dmc1*^*+/+*^ mice to address step-by-step differences in the recombination pathway as outlined below.

**Figure 3.**
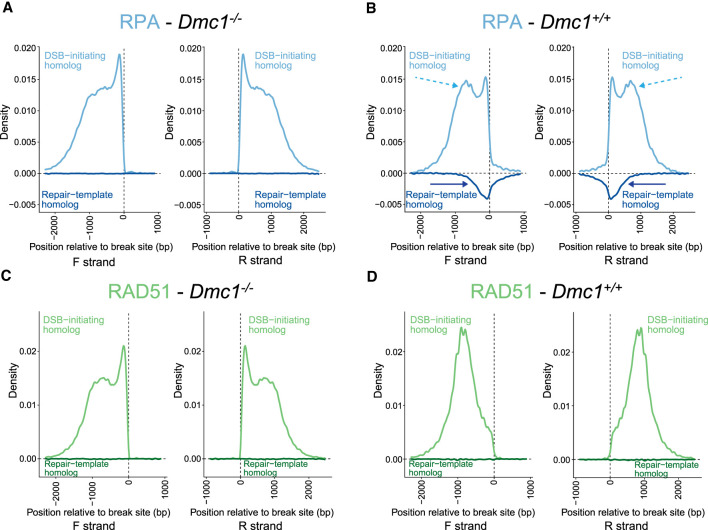
RPA and RAD51 binding in vivo in male mouse meiosis at asymmetric hotspots. (*A*) RPA binding to the DSB-initiating (*above* the *x*-axis) and repair template (*below* the *y*-axis) chromosomes in *Dmc1*^−/−^ (*left* two plots; forward [F] and reverse [R] strands, respectively) and (*B*) *Dmc1*^*+/+*^ (*right* two plots; F and R strands, respectively) male mice (see also Fig. 1A). Dashed light blue and dark blue arrows highlight differences in binding between the wild type and mutant on the two homologs. (*C*,*D*) As in *A*,*B* but for RAD51.

Nascent ssDNA overhangs are generated via resection after induction of DSBs (Paiano et al. 2020; Yamada et al. 2020), which are thought to be bound by RPA (Fig. 1A, steps 3–4). Consistent with this view, RPA binds the DSB-initiating chromosome in *Dmc1*^−/−^ to the left of the break on the forward (F) strand and to the right of the break on the reverse (R) strand (Fig. 3A). RPA binding is highest closest to the break-site and decreases with distance from it. This is consistent with RPA binding the full extent of resected ssDNA, in line with previously observed variation in the extent of DNA resection (Paiano et al. 2020; Yamada et al. 2020). This contrasts with the binding pattern of RPA on the DSB-initiating chromosome in *Dmc1*^*+/+*^ mice, which have a second peak 700–800 bp from the break site (Fig. 3B, dashed arrows). This suggests that there is additional DMC1-dependent RPA binding on the DSB-initiating chromosome in the wild type, and we address this further below.

RPA is thought to promote the formation of nucleoprotein filaments composed of RAD51 and DMC1 in wild-type meiosis (Fig. 1A, step 5). We showed previously that RAD51 and DMC1 have distinct localization in these filaments, with DMC1 binding closer to the break site and RAD51 binding closer to the junction with dsDNA in *Dmc1*^*+/+*^ mice (Fig. 3D; Hinch et al. 2020). In contrast, RAD51 binds the entire ssDNA filament in *Dmc1*^−/−^ testes (Fig. 3C). This suggests that DMC1 competes with or otherwise inhibits RAD51 filament formation close to the break site in wild-type meiosis, consistent with previous in vitro observations (Crickard et al. 2018).

In the wild type, DMC1- and RAD51-bound nucleoprotein filaments invade the homolog and engage with one of its strands via complementary base-pairing (Fig. 1A, steps 5–6). The other strand of the homolog becomes single-stranded and is stabilized by RPA in the so-called D-loop recombination intermediate (Hunter and Kleckner 2001; Hinch et al. 2020). This is evident in RPA binding to the repair-template chromosome in *Dmc1*^*+/+*^ mice (Fig. 3B, dark blue arrows). In contrast, we observe no signal whatsoever for RPA on the repair-template chromosome in *Dmc1*^−/−^ mice, showing that inter-homolog recombination intermediates are absent in the absence of DMC1. *Dmc1*^−/−^ mice are known to be defective in strand exchange (Pittman et al. 1998; Yoshida et al. 1998), and these data add to the understanding of their phenotype, showing no detectable fine-scale pairing in these mice.

**Figure 4.**
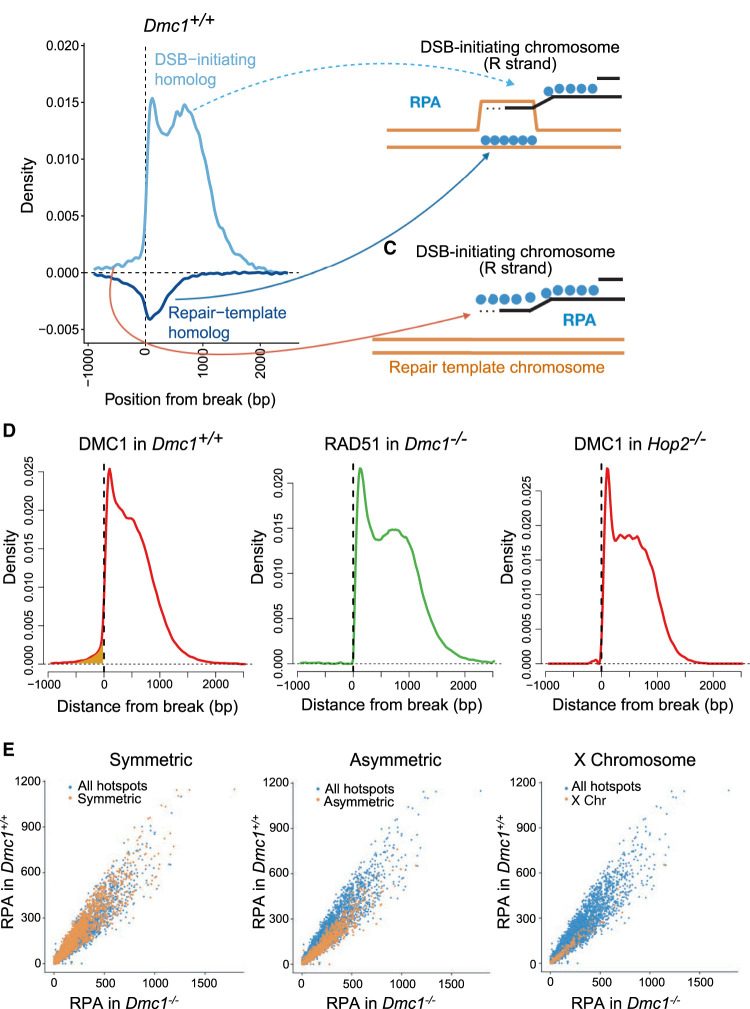
Properties of recombination intermediates. (*A*) RPA binding in *Dmc1*^*+/+*^ (as in Fig. 2B, but labeled with inferred recombination intermediates). (*B*) Model for RPA binding after successful strand invasion. RPA binds the DSB-initiating chromosome (black) and the repair-template chromosome (orange) with distinct localization. The dotted lines indicate newly synthesized DNA. (*C*) Model for RPA binding to the DSB-initiating chromosome subsequent to some DNA resynthesis. (*D*) Comparison of recombinase (DMC1/RAD51) binding on the wrong side of the break in animals proficient for strand invasion (*Dmc1*^*+/+*^) relative to those defective for strand invasion (*Dmc1*^−/−^ and *Hop2*^−/−^). The signal is highlighted in gold. *Hop2*^−/−^ data are from Khil et al. (2012). (*E*) Comparison of the estimated intensity of RPA binding (number of ChIP-seq reads within a peak corrected for background read frequency) in hotspots in *Dmc1*^−/−^ (*x*-axis) and *Dmc1*^*+/+*^ (*y*-axis) in symmetric, asymmetric, and nonpseudoautosomal X Chromosome hotspots, respectively.

RPA binding on the repair-template and the second peak in the binding profile of RPA on the DSB-initiating chromosome in the wild type (Fig. 3B, dark blue and light blue dotted arrows, respectively) are both dependent on DMC1. A natural and parsimonious explanation for this second peak is that RPA binds the DSB-initiating chromosome again *after* DMC1 and RAD51 dissociate from it following successful strand invasion (Fig. 4A,B). Under this model, RPA binds a segment of the resected DSB-initiating chromosome that is not part of the D-loop in addition to the repair-template chromosome (Fig. 4B). This interpretation is compatible with findings from electron micrography, which show bright foci of RPA at break sites after removal of DMC1 and RAD51 (Moens et al. 2002).

In addition to binding resection tracts, there is some RPA binding to the DSB-initiating chromosome that has the “wrong” polarity in *Dmc1*^+/+^ mice (Fig. 4A, vermillion arrow; Hinch et al. 2020). This binding is to the right of the break on the forward strand and to the left of the break on the reverse strand. These regions reflect segments of DNA that are resected and unavailable for binding on the DSB-initiating chromosome (Fig. 4A,C), and the reason for this signal was not previously clear. We note that this signal is not present in *Dmc1*^−/−^ mice (Fig. 3A), which indicates that it represents an unknown recombination intermediate that is abolished in the absence of DMC1. Two possible models may explain this presence of RPA with unexpected polarity on the DSB-initiating chromosome: One possibility is that it represents DNA that is newly synthesized in the D-loop (Fig. 4C). The repair of breaks is thought to proceed via recapture or reannealing of the extended strand to form crossover or noncrossovers, respectively (Marsolier-Kergoat et al. 2018), and RPA promotes both reactions by stabilizing the extended strand (Petalcorin et al. 2006; Sugiyama et al. 2006). Another, not mutually exclusive, possibility is that this intermediate is a distinct D-loop, not with the homolog but with the sister chromatid.

Note that, as with RPA, there is some DMC1 signal with the wrong polarity in wild-type mice (Fig. 4D, highlighted in gold; Hinch et al. 2020). To test whether this signal is also dependent on strand exchange, we compared the binding pattern of DMC1 in the wild type with another mutant defective in strand exchange (*Hop2*^−/−^) (Khil et al. 2012). We observed that, similar to RPA, DMC1 binding is abrogated in this region in the absence of strand exchange (Fig. 4D). The second model alone, namely, a D-loop with a sister chromatid, cannot explain the presence of DMC1 with wrong polarity as DMC1 does not bind ssDNA in a D-loop (Hinch et al. 2020) (DMC1-bound DNA in a D-loop in double-stranded). However, the data are compatible with the first model in which DMC1 binds newly synthesized DNA after strand exchange. Under this model, the presence of DMC1 on newly synthesized DNA can be explained by stable strand exchange proceeding via multiple rounds of DMC1 loading, strand invasion, and template switching in mouse, as observed in *S. cerevisiae* (Marsolier-Kergoat et al. 2018).

Little is known about the dynamics and lifespans of strand-exchange intermediates in mammals, although our previous work showed that intermediates destined for crossover events are likely to have longer lifespans than those destined for noncrossovers in the mouse (Hinch et al. 2020). As discussed above, RPA binds recombination intermediates *before* strand exchange in both *Dmc1*^−/−^ and wild-type mice but *after* strand exchange only in wild-type mice. Comparison of RPA binding signal in *Dmc1*^−/−^ and wild-type mice is thus informative about post-strand-exchange intermediates. Previous work has shown that breaks in symmetric hotspots are more likely to be repaired with the homologous chromosome than are DSBs in asymmetric hotspots (Hinch et al. 2019; Li et al. 2019). Breaks in asymmetric hotspots that remain unrepaired at a late stage in pachytene are thought to be repaired using the sister chromatid as template. Similarly, breaks on the nonpseudoautosomal X Chromosome lack a homolog and are thought to be repaired using the sister chromatid. We assessed the difference in lifespans in inter-homolog and inter-sister strand-exchange intermediates by comparing the signal of RPA signal in these sets of hotspots (Fig. 4E). We observe that symmetric hotspots have a stronger signal of RPA binding on average than asymmetric or X Chromosome hotspots in *Dmc1*^*+/+*^ relative to *Dmc1*^−/−^. This difference cannot be explained by the extent of resection or the size of the D-loop (Supplemental Fig. S5). In contrast, these data are consistent with a model in which the average lifespans of inter-homolog strand-exchange intermediates are longer than those of inter-sister intermediates in the wild type. A natural explanation is that once the barrier to sister repair is removed in late pachytene (Lao and Hunter 2010; Lu and Yu 2015; Li et al. 2019), breaks are repaired rapidly, on average. However, we cannot rule out that there is a physiological difference between inter-homologous and inter-sister intermediates that impacts their detection.

## Discussion

Our data extend our understanding of the role of DMC1 in mammalian meiosis, by using F_1_ hybrid mice with and without this key protein. Meiotic recombination involves interaction between the chromosome on which a break has occurred with the chromosome that is used as template for repair. Use of an F_1_ hybrid enables us to distinguish these distinct processes, revealing the molecular structures of intermediates involved in meiotic DNA break repair. Although the absence of stable strand exchange has been shown cytologically in *Dmc1*^−/−^ mice, these data show at a molecular level that strand-exchange intermediates are absent in these animals. We also find that the pattern of RAD51 binding in relation to the break site is altered in the absence of DMC1. We showed previously that RAD51 coats only distal to the break site in wild-type mice (Hinch et al. 2020). In contrast, ssDNA is bound both proximally and distally to the break by RAD51 in the absence of DMC1. We have also obtained further insight into the structure of D-loop intermediates in wild-type meiosis, with evidence for RPA binding both the DSB-initiating and repair-template chromosomes following successful strand invasion. Furthermore, our data are suggestive of DMC1 and RPA binding newly synthesized DNA before resolution with a crossover or noncrossover, possibly reflecting multiple rounds of strand invasion and extension. Finally, our data suggest that RPA-bound inter-homolog intermediates have a longer lifespan on average than inter-sister intermediates. These insights are inferred from changes in protein binding between mutant and wild-type F_1_ mice and the majority of them would have been invisible in equivalent inbred organisms. These data thus show the benefit of using hybrid model organisms to understand recombination.

Generating hybrid knockout mice using conventional breeding of pre-existing lines involves breeding schemes of considerable size and complexity. In contrast, performing mutagenesis directly in mice with a hybrid background enables relatively high-throughput functional analysis of candidate recombination genes. This can be achieved without extensive breeding, reducing both the numbers and costs of animals used. Although founder analysis is widely used for functional studies in zebrafish (Shah et al. 2015; Wu et al. 2018), use of this technique is less common in functional studies using mice (Kroll et al. 2021). One concern of this approach is mosaicism (Teboul et al. 2017), which can result from the introduction of CRISPR-Cas9 nucleases into the early embryo and their persistence past the first few cleavage events (Yen et al. 2014; Oliver et al. 2015). Our approach mitigates this concern by thorough validation of the target protein ablation in the tissue of interest. Further, the knockout mutations generated by the activity of these nucleases are often variable between individual founder mice. A diversity of knockout alleles, all conferring a consistent phenotype, conveys robustness to the experimental design. We believe that our approach has considerable power for enabling a detailed functional characterization of proteins involved in meiotic recombination, achievable at low animal cost.

## Methods

### Animals

All animal procedures were performed in accordance with UK Home Office Animal (Scientific Procedures) Act 1986, with procedures reviewed by the clinical medicine animal welfare and ethical review body at the University of Oxford and conducted under project license PAA2AAE49. Animals were housed in individually ventilated cages, provided with food and water ad libitum, and maintained on a 12-h/12-h light–dark cycle (150–200 lux). The only reported positives on FELASA health screening over the entire time course of these studies were for *Entamoeba* spp. Experimental groups were determined by genotype and were therefore not randomized, with no animals excluded from the analysis. Sample sizes for ChIP analysis were selected on the basis of previously published studies (Hinch et al. 2020), and all phenotypic characterization was performed blind to experimental group.

Genetically modified mice harboring the human PRDM9 B allele (*Prdm9*^*tm1.1(PRDM9)Wthg*^) were generated inhouse (Davies et al. 2016). CAST/EiJ mice were provided by Jonathon Godwin at the Sir William Dunn School of Pathology, University of Oxford. C57BL/6J mice were purchased from Charles River Laboratories.

### Design and in vitro assessment of sgRNA activity

sgRNAs were designed against the relevant portions of the *Dmc1* genomic sequence (Supplemental Fig. S4A; Supplemental Table S4) using the CRISPOR algorithm (Haeussler et al. 2016) and were synthetized (Synthego). sgRNA were tested for activity by lipofection (RNAiMax, Invitrogen) into a mouse melanocyte cell line (B16F10), pre-engineered to stably express the Cas9 nuclease, followed by incubation for 48 h, and then pooled genomic DNA was assessed for the presence of indel mutations by Sanger sequencing and quantified using the tracking of indels by decomposition (TIDE) algorithm (Brinkman et al. 2014).

### Genetic modification

Wild-type C57BL/6J females or C57BL/6J females homozygous for the *Prdm9*^*tm1.1(PRDM9)Wthg*^ allele were superovulated at 3 wk of age and mated with either wild-type C57BL/6J males or CAST/EiJ males. Fertilized zygotes in batches of up to 100 were electroporated in Opti-MEM media containing either 65 ng/µL of both the 5′ and the 3′ sgRNA and 650 ng/µL recombinant NLS-Cas9 protein (two square-wave pulses of 30 V, 3-msec duration, and 100-msec interval) using a GenePulser Xcell electroporator (Bio-Rad) and a 1-mm electrofusion slide (BLS GSS-1000). Electroporated zygotes were cultured overnight to the two-cell stage and surgically implanted into recipient pseudopregnant CD1 females. The production data for the founder mice are summarized in Supplemental Table S3. Offspring were genotyped using PCR amplicons spanning the CRISPR-Cas9 target sites (Supplemental Table S4) followed by Sanger sequencing. An assessment of the degree of mutagenesis was performed using the TIDE algorithm (Brinkman et al. 2014).

### Phenotyping of mice

Mice were sacrificed at 10 wk of age. Paired testis weight from individual mice was recorded and normalized against lean body weight, as assessed using an EchoMRI-100 small animal body composition analyzer. Sperm count was obtained from individual mice by allowing caudal epididymis sperm to swim out in 1000 μL of warm PBS before counting with a hemocytometer.

Testis pairs from each individual putative founder male were processed by removing the tunica, and one-quarter of the material was prepared for surface spreading and immunostained as previously described (Davies et al. 2016). The following primary antibodies were used: mouse polyclonal anti-SYCP3 (Santa Cruz Biotechnology sc-74569, D-1), biotinylated rabbit polyclonal anti-SYCP3 (Novus Biologicals NB300-232), rabbit polyclonal anti-SYCP1 (Novus Biologicals NB300-229), rabbit polyclonal anti-RPA2 (Abcam ab10359), and rabbit polyclonal anti-DMC1 (H-100; Santa Cruz Biotechnology sc-22768) and rabbit polyclonal anti-DMC1 (Proteintech 13714-1-AP). Detection was performed with Alexa Fluor 594- or 488-conjugated secondary antibodies against rabbit or mouse IgG, respectively (Thermo Fisher Scientific), and Cy5-labeled streptavidin (Thermo Fisher Scientific) for the biotinylated antibody detection. Images were acquired using either a BX-51 upright wide-field microscope equipped with a JAI CVM4 B&W fluorescence CCD camera and operated by Leica Cytovision Genus software, or a Leica DM6B microscope for epifluorescence equipped with a DFC 9000Gt B&W fluorescence CCD camera and operated via the Leica LASX software. Image analysis was performed using Fiji (ImageJ-win64).

The remaining three-quarters of the testis material was snap-frozen and stored at −80°C until confirmation of *Dmc1* knockout status was ascertained from the above immunohistochemical staining with a DMC1 antibody. Only founder mice that showed no detectable staining of DMC1 at zygotene stage were used for ChIP-seq experiments and in the analysis of testis weights and sperm counts.

### RPA and RAD51 ChIP-seq maps

ChIP followed by SSDS was performed as described previously (Khil et al. 2012), with modifications (Hinch et al. 2020). The RAD51 assay was sequenced on an Illumina HiSeq 2500 sequencer with 51-bp paired-end reads. The RPA assay was sequenced on an Illumina HiSeq X sequencer with 150-bp paired-end reads. The reads were trimmed to 75 bp before processing with the analytical pipeline to identify ssDNA (Khil et al. 2012). Hotspots were called using our published peak-calling algorithm (Davies et al. 2016). Supplemental Table S5 summarizes the characteristics of the experimental data sets.

For each hotspot, we also inferred the fraction of reads that originated from the B6 and the CAST chromosomes respectively, as previously described (Davies et al. 2016). PRDM9 has a histone methyltransferase activity, and binding to its target sites is associated with trimethylation of H3K4. Using this histone mark as a surrogate for PRDM9 binding enables H3K4me3 ChIP-seq data to be used to infer whether the binding of PRDM9 occurs equally on both parental chromosomes of the hybrid (these hotspots are referred to as being symmetric) or whether there binding occurs preferentially to one or other of the parental chromosomes (these hotspots are referred to as being asymmetric) (Hinch et al. 2019). For the purpose of this analysis, asymmetric hotspots were defined as hotspots wherein the fraction of H3K4me3 reads originating on the B6 Chromosome, denoted *f*, was either *f* ≥ 0.9 or *f* ≤ 0.1, as previously described (Hinch et al. 2019). The plots shown inferred protein binding relative to a DSB site through deconvolution, as previously described (Hinch et al. 2019), with a small modification: We used the glmnet R package (Friedman et al. 2010) instead of the ginv R package to enforce a nonnegativity constraint in the linear optimization. The control *Dmc1*^+/+^ data are from Hinch et al. (2020). Material from three individual *Dmc1*^−/−^ founders were pooled for the ChIP-seq analysis data: Mice with IDs 8.1a, 9.1a, and 9.1b were pooled for the RPA2 data, and mice with IDs 3.1a, 3.1c, and 4.1b were pooled for the RAD51 data (see Table 1). The following antibodies were used for the ChIP-seq data: mouse monoclonal anti-RPA2 (Calbiochem RPA34-20) and mouse monoclonal anti-Rad51 (Novus Biologicals 14B4).

### Data sets

Previously published data sets used in this study are listed in Supplemental Table S4C.

## Data access

All raw and processed sequencing data generated in this study have been submitted to the NCBI Gene Expression Omnibus (GEO; https://www.ncbi.nlm.nih.gov/geo/) under accession number GSE239997.

## Supplementary Material

Supplement 1

Supplement 2

Supplement 3

Supplement 4

Supplement 5

Supplement 6

Supplement 7

Supplement 8

Supplement 9

Supplement 10
