# Supplementary material for: Characterization of meiotic recombination intermediates through gene knockouts in founder hybrid mice
Source: Genome Res. 2023 Nov;33(11):2018–27. doi: 10.1101/gr.278024.123 (PMC10760447; doi:10.1101/gr.278024.123)
Supplement: Supplement 1 [file Supplemental_Fig_S1.pdf]

A

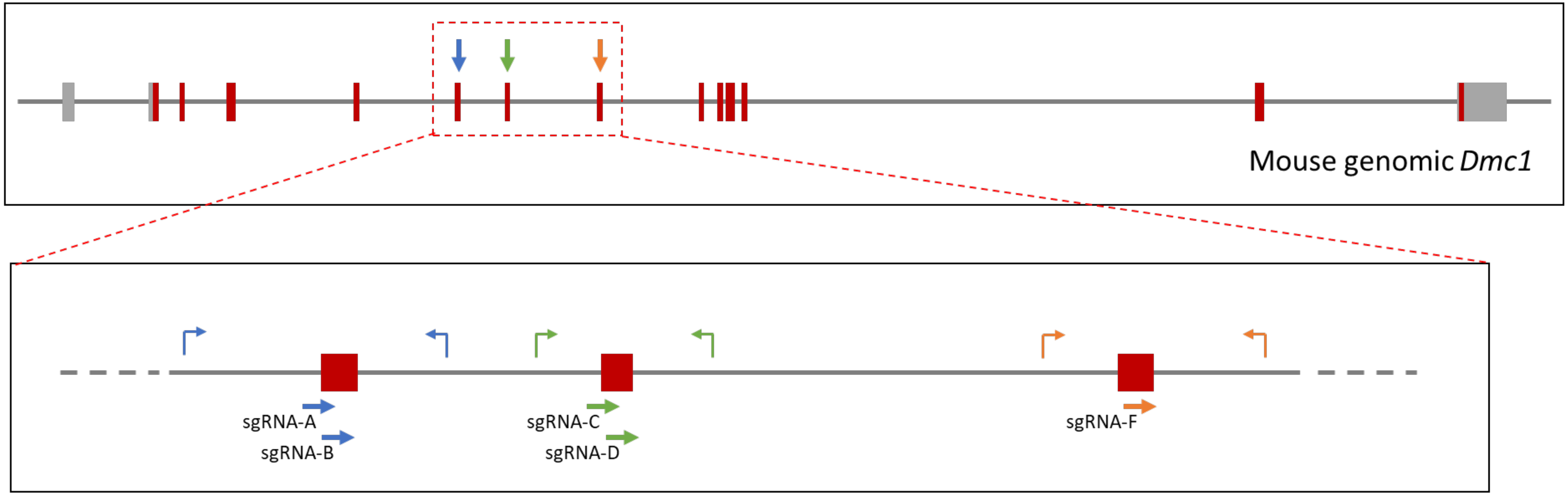

B

sgRNA	Target sequence (5'-3')	Exon to be targeted	In vitro cutting efficiency (TIDE)
sgRNA-A	GTTTTCAGTAAATTACTAGG AGG	6	81%
sgRNA-B	AGGAGGTGGCATTGAAAGTA TGG	6	8%
sgRNA-C	AATTTCACAGAATTTCGTAC TGG	7	1%
sgRNA-D	CAGCTGTCTCATACCCTCTG TGG	7	25%
control	GATGGTGATGGTGCGCCAGG AGG	N/A	62%

C

sgRNA	Target sequence (5'-3')	Exon to be targeted	In vitro cutting efficiency (TIDE)
sgRNA-F	CCAGGAACAGGCGGCTACTC AGG	8	89%
control	GATGGTGATGGTGCGCCAGG AGG	N/A	64%

Supplementary Figure S1. Production details for generation of hybrid *Dmc1*$^{-/-}$ mice. (A) Genomic structure of the *Dmc1* gene showing the positions of the sgRNAs. (B)(C) Sequences and in vitro cutting efficiencies of the selected sgRNAs.
