# Supplementary material for: Characterization of meiotic recombination intermediates through gene knockouts in founder hybrid mice
Source: Genome Res. 2023 Nov;33(11):2018–27. doi: 10.1101/gr.278024.123 (PMC10760447; doi:10.1101/gr.278024.123)
Supplement: Supplement 2 [file Supplemental_Fig_S2.pdf]

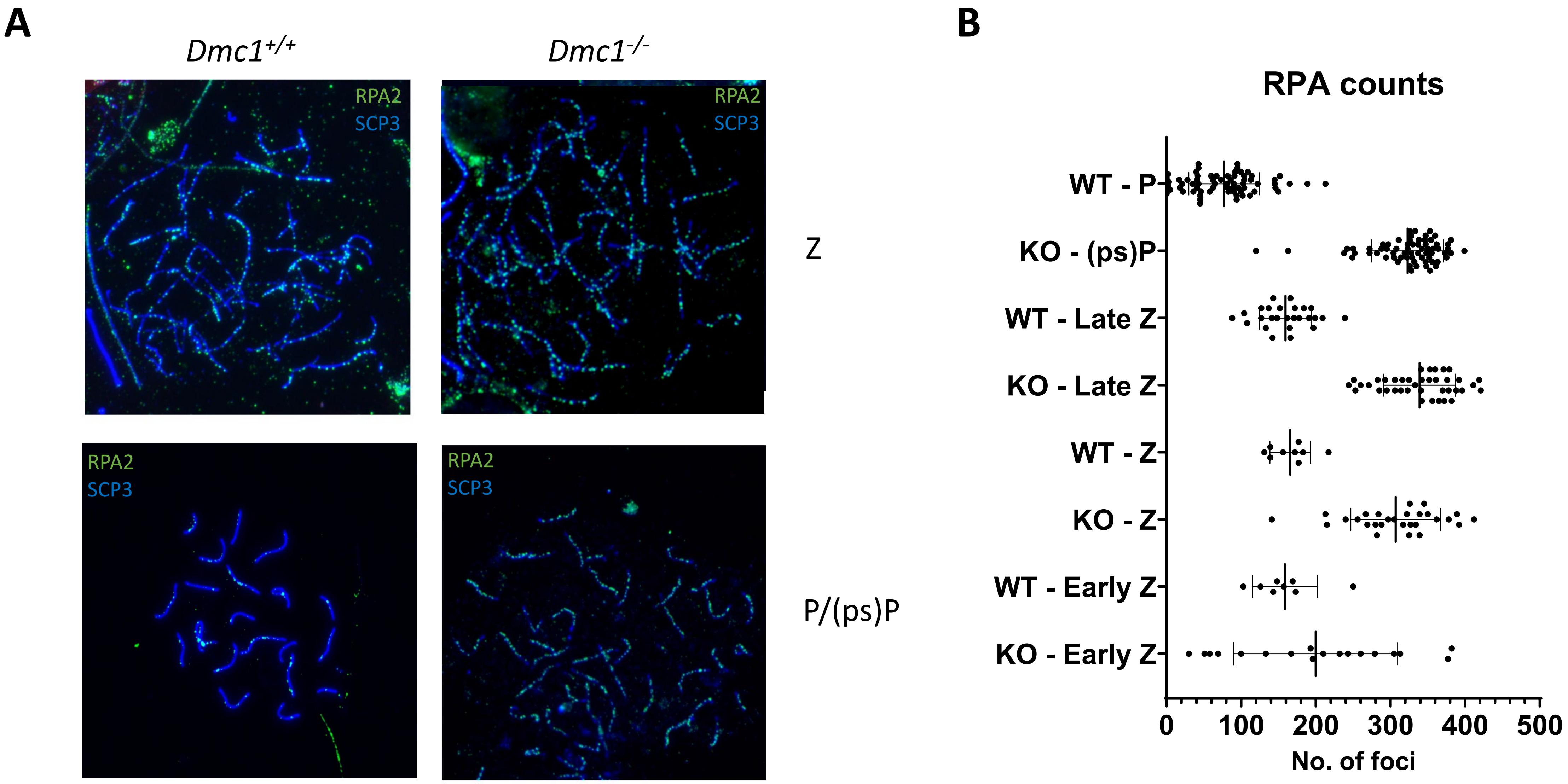

Supplementary Figure S2. RPA2 localization and foci counts in *Dmc1* Knockout hybrid founders. (A) Representative immunofluorescence staining of RPA2 (green) in testis nuclear spreads from founder mice, one with DMC1 present (*Dmc1*$^{+/+}$) and ablated (*Dmc1*$^{-/-}$) with co-staining of the synaptonemal complex protein SYCP3 which labels the chromosome axis (blue) in zygotene (Z) (top panels) and pachytene(P)/pseudopachytene ((ps)P) (middle panels). (B) RPA2 foci counts in *Dmc1*$^{+/+}$ (WT) and *Dmc1*$^{-/-}$ (KO) zygotene (early, mid and late) and pachythene/psuedopachytene.
