# Supplementary material for: Characterization of meiotic recombination intermediates through gene knockouts in founder hybrid mice
Source: Genome Res. 2023 Nov;33(11):2018–27. doi: 10.1101/gr.278024.123 (PMC10760447; doi:10.1101/gr.278024.123)
Supplement: Supplement 3 [file Supplemental_Fig_S3.pdf]

A

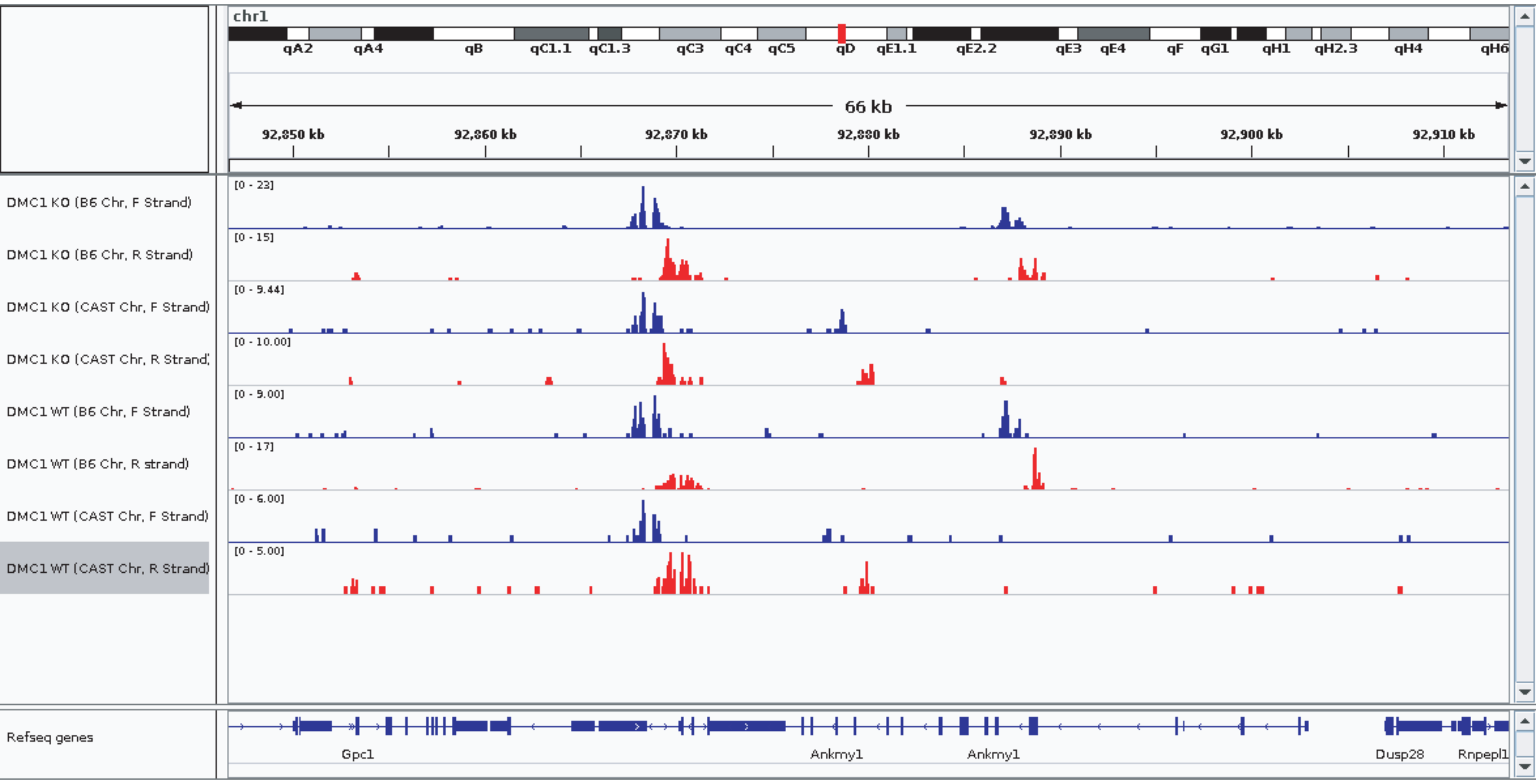

B

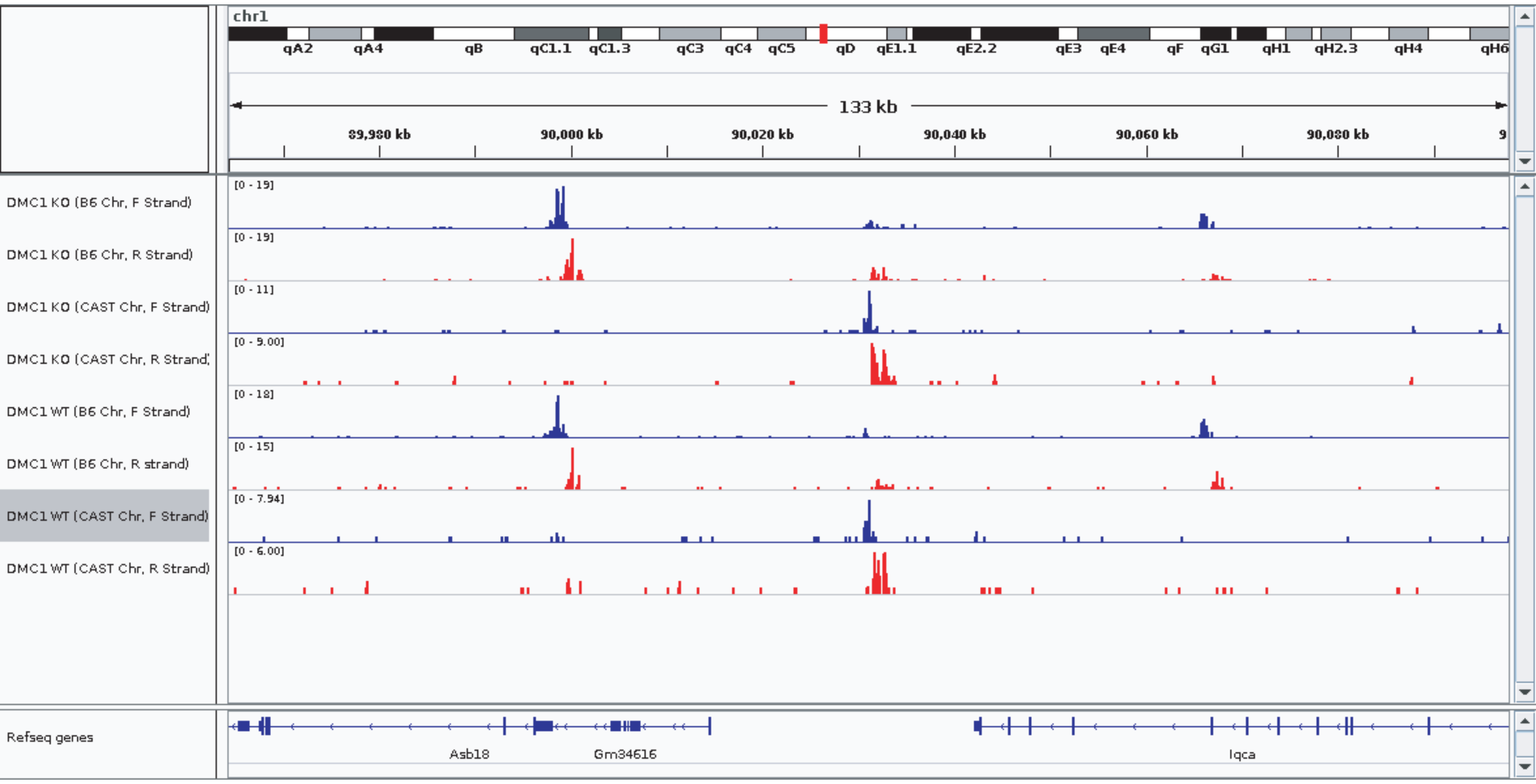

Supplementary Figure S3. Integrative Genomics Viewer (IGV) plots illustrating RAD51 ChIP-seq read-depth at two distinct loci (A, B). The top four tracks show depth of forward and reverse strand reads mapping to the B6 chromosome and CAST chromosomes respectively in *Dmc1*$^{-/-}$ (KO) mice. The bottom four tracks show the corresponding values assayed in *Dmc1*$^{+/+}$ (WT) mice. Three hotspots were identified in each of these loci. Some of the hotspots have comparable signal on both chromosomes (symmetric hotspots) whilst others have reads from predominantly one chromosome (asymmetric hotspots).
