# Supplementary material for: Characterization of meiotic recombination intermediates through gene knockouts in founder hybrid mice
Source: Genome Res. 2023 Nov;33(11):2018–27. doi: 10.1101/gr.278024.123 (PMC10760447; doi:10.1101/gr.278024.123)
Supplement: Supplement 4 [file Supplemental_Fig_S4.pdf]

A

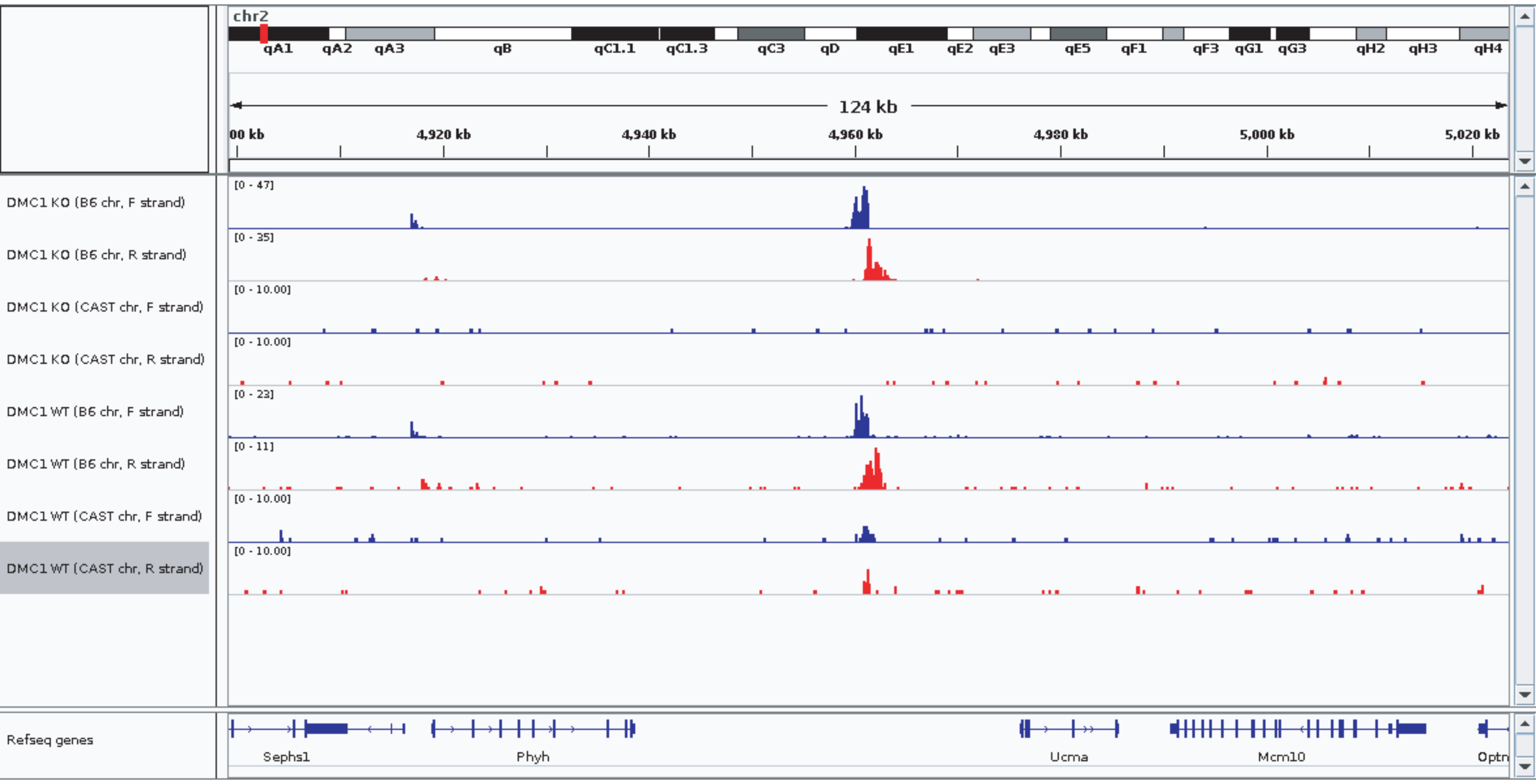

B

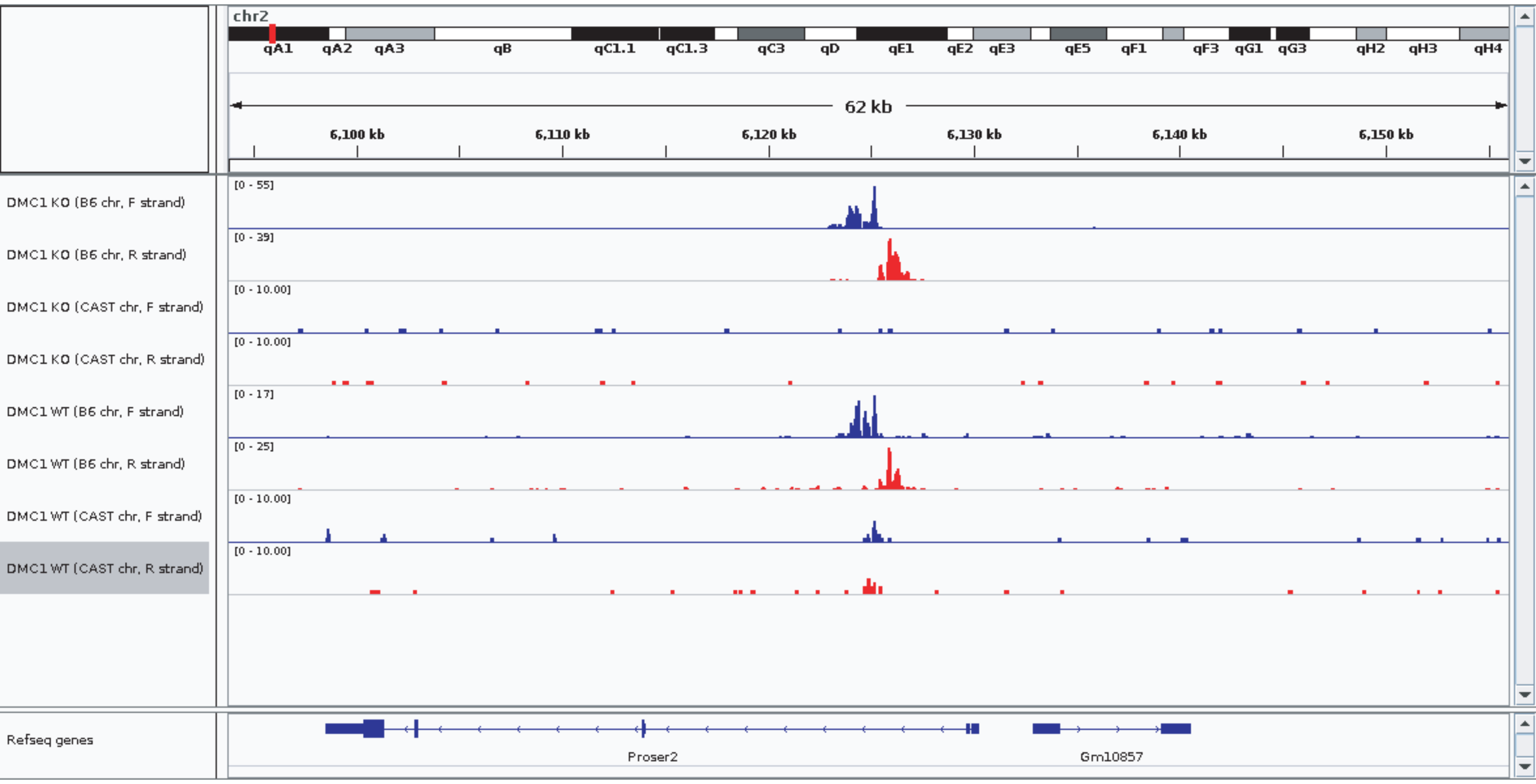

Supplementary Figure S4. Integrative Genomics Viewer (IGV) plots illustrating RPA ChIP-seq read-depth in two hotspots (A, B). The top four tracks show depth of forward and reverse strand reads mapping to the B6 chromosome and CAST chromosomes respectively in *Dmc1*$^{-/-}$ (KO) mice. The bottom four tracks show the corresponding values assayed in *Dmc1*$^{+/+}$ (WT) mice. Both hotspots are activated by the PRDM9 CAST allele, which binds the B6 chromosome more strongly in these hotspots. Note that the B6 chromosome has RPA signal in both *Dmc1*$^{-/-}$ and *Dmc1*$^{+/+}$ mice, while the CAST chromosome has signal only in *Dmc1*$^{+/+}$ mice.
