# Supplementary material for: Characterization of meiotic recombination intermediates through gene knockouts in founder hybrid mice
Source: Genome Res. 2023 Nov;33(11):2018–27. doi: 10.1101/gr.278024.123 (PMC10760447; doi:10.1101/gr.278024.123)
Supplement: Supplement 5 [file Supplemental_Fig_S5.pdf]

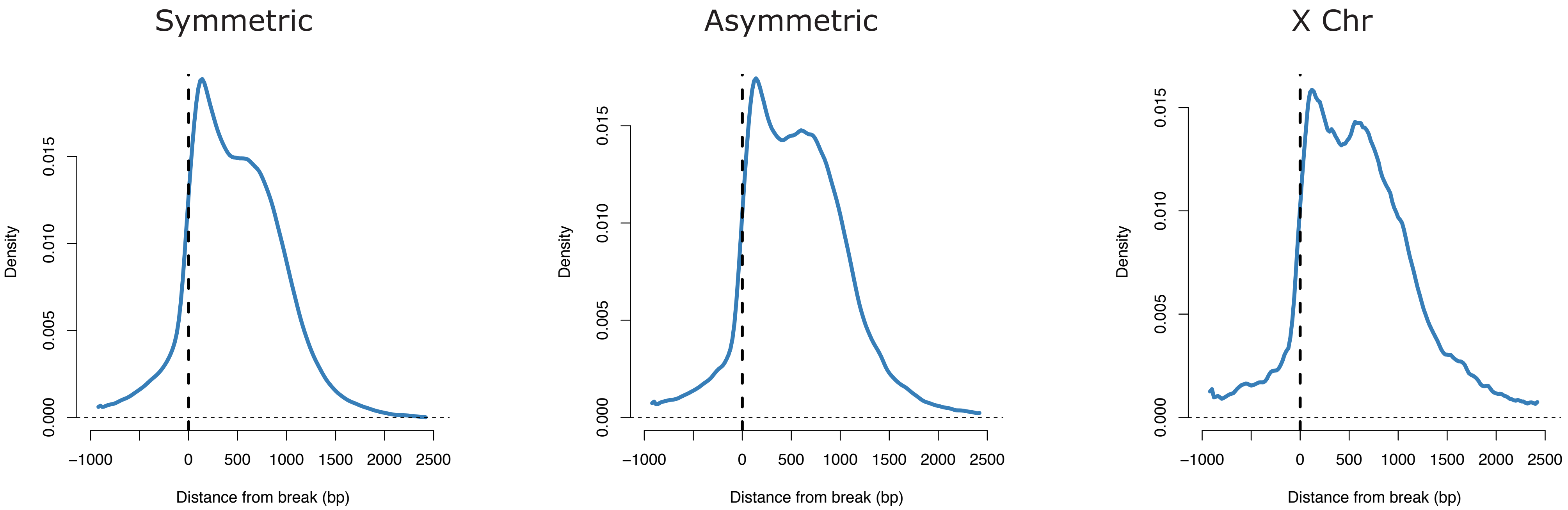

Supplementary Figure S5. Footprint of RPA binding in symmetric, asymmetric and X Chromosome hotspots in *Dmc1*$^{+/+}$ mice (R strand shown). The data do not support greater resection (right of the break site) or D-loop extent (left of the break site) in symmetric hotspots.
